# A Case of Emphysematous Abscess of Thigh, from Intestinal Injury

**Published:** 1886-09

**Authors:** G. Herbert Lilley

**Affiliations:** Medical Officer, H.M. Convict Prison, Portland


### A CASE OF EMPHYSEMATOUS ABSCESS OF THIGH, FROM INTESTINAL INJURY. By G. HERBERT LILLEY, M.D., Medical Officer, H.M. Convict Prison, Portland.

J. B., male, age 54, was admitted to the hospital of this prison on August 30th, 1883, with crepitating swelling, involving the subcutaneous areolar tissue of nearly the whole of the front of abdomen, extending to chest and back, and to inner side of both thighs. He stated he had been swollen for some time, but he did not think it worth noticing, until it caused personal inconvenience. I ascertained that, about five years before admission, he was struck upon the left groin by a heavy piece of falling timber, and that, on January 17th, 1882, he discovered that he was ruptured on the left side. An ordinary truss for oblique inguinal hernia was supplied. He remained in hospital until October 5th, 1883, when a flannel roller was applied to his chest and abdomen, &c., and he was put to light work,—emphysema at the time being confined only to lower abdominal region, and slightly to thighs. He was periodically examined till October 7th, 1884, when it was found that there was present a considerable degree of inflammation of inner side of left thigh. From the man's account, the redness developed "in a few hours." I painted a broad band of demarcation with nitrate of silver above and below area of inflammatory redness, and poulticed the limb. On the 11th of October an abscess burst on outer side of thigh, and about five ounces of most offensive pus escaped. A few hours afterwards an opening formed at middle of front of thigh, through which five or six ounces of fæcal fluid were evacuated. I syringed well with carbolic acid lotion (1 in 20) through the openings, and made a counter-opening, inserting a carbolised rubber drainage tube. The wounds were dressed antiseptically, under steam spray. An opiate gave a fairly good night's rest. On the following day a large slough detached from inner side of Scarpa's triangle, followed by the escape of four ounces of fæcal fluid. On the 13th, the discharge from all the openings was less offensive, and tenderness of limb diminished. After well syringing as before, I applied dressings of boracic acid and vaseline (Ferris) on boric lint. On the 15th there was marked improvement, although some bagging was present above the knee. By well bandaging the knee, and elevating the limb, free drainage removed the bagging. On the 16th I removed the rubber tube, and syringed well with carbolic acid lotion (1 in 40), the sinuses looking healthy. On 17th, diarrhœa set in. On 20th, diarrhœa continuing, a circumscribed blush appeared on inner side of thigh. Argent. nit. was again employed, and the redness encircled. On the 26th an abscess burst at this spot, the pus being only slightly offensive. I again syringed with the stronger solution of carbolic acid. Diarrhœa appeared to resist all treatment till the 31st of October, when it ceased. On November 2nd, the wounds had all closed. On the 15th, he could sit up half the day in his chair. On November 25th I discharged him from hospital: all emphysema was gone, and walking caused no pain or inconvenience. He was transferred on this date to a milder climate, with a gain in weight of 20 lb. during the seven weeks he was under treatment in hospital.

